# Highly Pathogenic Avian Influenza A(H5N1) Virus in a Harbor Porpoise, Sweden 

**DOI:** 10.3201/eid2904.221426

**Published:** 2023-04

**Authors:** Elina Thorsson, Siamak Zohari, Anna Roos, Fereshteh Banihashem, Caroline Bröjer, Aleksija Neimanis

**Affiliations:** National Veterinary Institute (SVA), Uppsala, Sweden (E. Thorsson, S. Zohari, F. Banihashem, C. Bröjer, A. Neimanis);; Swedish Museum of Natural History, Stockholm, Sweden (A. Roos)

**Keywords:** influenza A virus, H5N1 subtype, pathology, wild animals, Phocoena, porpoises, mammals, meningitis/encephalitis, influenza in birds, viruses, Sweden, influenza, zoonoses

## Abstract

We found highly pathogenic avian influenza A(H5N1) virus clade 2.3.4.4b associated with meningoencephalitis in a stranded harbor porpoise (*Phocoena phocoena*). The virus was closely related to strains responsible for a concurrent avian influenza outbreak in wild birds. This case highlights the potential risk for virus spillover to mammalian hosts.

Europe and, more recently, the Americas are experiencing unprecedented mortality in wild and domestic birds because of the highly pathogenic avian influenza virus (HPAI) A(H5N1) virus clade 2.3.4.4b (*1*). Infections in tens of thousands of wild birds representing at least 112 species, including large numbers of seabirds, have been documented (*1*,*2*). Since December 2021, HPAI H5N1 has dominated infections in wild birds in Sweden, other countries in Europe, and the Americas, and it has spilled over into wild mammals, such as red foxes and mustelids (*1*). Increased mortality in harbor seals (*Phoca vitulina*) and gray seals (*Halichoerus grypus*) in eastern North America has been associated with HPAI H5N1 infection (*3*). Although influenza A virus (IAV) infections in seals have been repeatedly documented, reports in cetaceans are scarce (*4*). We are aware of only 2 cases where IAV in a cetacean might have been associated with disease (*4*,*5*). We report HPAI H5N1 infection in a harbor porpoise (*Phocoena phocoena*) in Sweden.

In late June 2022, an immature male harbor porpoise became stranded in shallow water off the west coast of Sweden (58.64817 N, 11.28973 E). It swam in circles, was unable to right itself, and drowned shortly after discovery. The carcass was stored frozen until necropsy examination at the National Veterinary Institute (Uppsala, Sweden). Macroscopic findings were minimal and included pulmonary edema consistent with drowning. Microscopically, we detected moderate lymphoplasmacytic meningoencephalitis with neuronal necrosis, gliosis, perivascular cuffing, and vasculitis in the brain (Figure 1, panel A). The lung contained few areas of mild, mononuclear septal thickening and increased numbers of alveolar macrophages.

**Figure 1:**
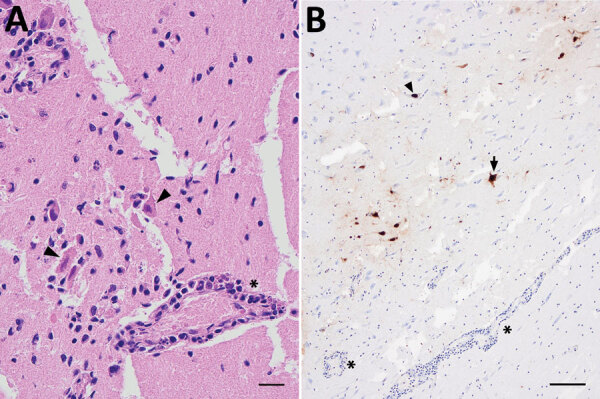
Microscopic analyses of tissue samples from a harbor porpoise (*Phocoena phocoena*) infected with highly pathogenic avian influenza virus H5N1 clade 2.3.4.4b, Sweden. A) Brain tissue showing neuronal necrosis (arrowheads) and perivascular lymphoplasmacytic cuffing of vessels and vasculitis (asterisk). Scale bar represents 20 µm. B) Immunohistochemical labeling of influenza A nucleoprotein in neuronal nuclei (arrowhead) and cytoplasm (arrow), as well as glial cells. Perivascular cuffing (asterisks) is seen in close association to influenza A immunolabeling. Scale bar represents 100 µm.

Stranded porpoises in Sweden are screened routinely for cetacean morbilliviruses, which can be neurotropic in cetaceans, and IAV, which can be neurotropic in other species (*4*,*6*). We analyzed pooled lung, spleen, and brain samples for cetacean morbilliviruses by using real-time reverse transcription PCR (*6*) with the addition of an in-house designed hydrolysis probe (6FAM-TGG TTC CAA CAG GYA G-MGB) for detection. No morbilliviral RNA was found. We detected IAV genome from lung and bronchial swab specimens (*7*) and subtyped the virus as HPAI H5N1; viral genome sequences were determined directly from tissue samples. Phylogenetic analysis of the complete genome sequences (GISAID accession nos. EPI2150621–8) classified the virus as H5N1 clade 2.3.4.4b. The genome contained no genetic motif of mammalian adaptation besides those already described for H5 clade 2.3.4.4 (HA-H5 172A-Airborne transmission, M1 N30D, Y215A-Virulence// NS1 P42S, L103F and I106M-Virulence) (*8*). Detecting IAV in respiratory tract swab specimens prompted us to analyze other organs. We detected the highest IAV loads in the brain (cycle threshold [Ct] value 20.57) and smaller loads in the lungs (Ct 30.72), kidney (Ct 31.37), liver (Ct 32.75), and spleen (Ct 33.43). We detected no virus in the intestine, muscle, or blubber.

We performed immunohistochemical analysis using a commercial influenza A nucleoprotein primary monoclonal antibody (EBS-I-238; Biologicals Limited, https://biologicals-ltd.com) as previously described (*9*) on all organs containing IAV genome to examine viral antigen distribution and the relationship to pathological lesions. We noted multifocal areas of moderate immunolabelling in the brain in nuclei and cytoplasm of neurons (Figure 1, panel B), glial cells, and epithelial cells of the choroid plexus. Scant intranuclear and intracytoplasmic viral antigen was in scattered cells in alveoli (macrophages or sloughed epithelium). We did not observe any viral antigen in other tissues examined.

IAV infection in a harbor porpoise represents expanding viral host range. Infections in cetaceans can result in animal death, as demonstrated by the abnormal behavior and subsequent drowning caused by the meningoencephalitis associated with infection. Virus was found predominantly in the brain, a finding consistent with H5N1 clade 2.3.4.4b infection in other mammals (*10*). Routine examination of the brain is warranted in cetacean disease surveillance, and IAV infection should be considered in animals demonstrating abnormal behavior or neuropathology. The virus detected in this porpoise was most closely related to viruses circulating in wild birds at the same time and location (Figure 2), indicating likely spillover from wild birds. The route of transmission is unknown but includes contact with infected birds or indirect contact through contaminated water, suggesting that infection pressure in the ecosystem was high. 

**Figure 2:**
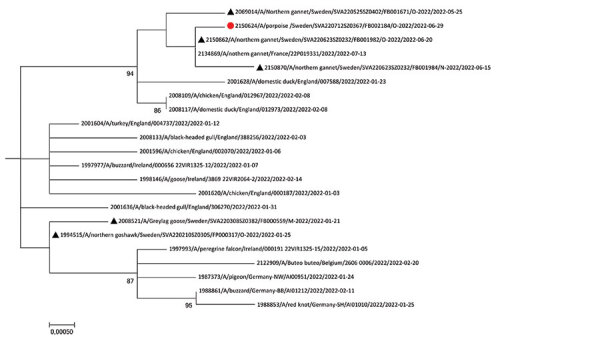
Maximum-likelihood phylogenetic tree for the hamagglutinin gene of highly pathogenic avian influenza A(H5N1) virus isolates from a harbor porpoise (red dot) and wild and domestic birds (black triangles) from Sweden and reference viruses. The number at each node represents the sequence accession number. Bootstrap values (2,000 replicates) >70% are displayed at the branch nodes. Scale bar indicates number of nucleotide substitutions per site.

Although the genome of the detected HPAI H5N1 virus did not contain any known genetic marker of mammal adaptation, the clinical manifestations and presence of virus in diverse organs, including the brain, indicate the potential risk of HPAI viruses to mammalian hosts even without adaptation. This risk is a consideration for persons in close contact with infected animals. In addition, extensive circulation of the HPAI H5Nx virus clade 2.3.4.4b in wild and domestic bird populations and sporadic transmission to humans and other mammals enables the virus to evolve, increasing the risk of it becoming more transmissible or pathogenic for mammals. Understanding the epidemiology and host–pathogen environmental ecology of IAVs among wildlife, coupled with continuous surveillance, developing better tools for risk assessments, and updating public and animal health countermeasures and intervention strategies, are essential for reducing the threats of zoonotic influenza.
